# Influence of High-Speed Ram Transition Position on Porosity and Mechanical Properties of Large One-Piece Die-Casting Al-Si-Mn-Mg Aluminium Alloy

**DOI:** 10.3390/ma17246169

**Published:** 2024-12-17

**Authors:** Sai Zhang, Pengfei Ren, Kangle Wang, Bo Liu, Xianming Meng

**Affiliations:** 1 China Automotive Technology and Research Center Co., Ltd., Tianjin 300300, Chinarenpengfei@catarc.ac.cn (P.R.); 2 School of Mechanical Engineering, University of Science and Technology Beijing, Beijing 100083, China; 3 Beijing Key Laboratory of Lightweight Metal Forming, University of Science and Technology Beijing, Beijing 100083, China

**Keywords:** high-pressure die-casting, aluminium alloy castings, process parameters, piston switching position, microstructure analysis, optimisation of casting structure

## Abstract

The high-pressure die-casting process can effectively manufacture aluminium alloy castings with complex shapes and thin wall thicknesses. However, due to the complex flow characteristics of the liquid metal during the mould-filling process, there are significant differences in the mechanical properties of different parts of the casting. This paper analyses the effect of the high-speed ram transition position on porosity and mechanical properties of Al-Si-Mn-Mg aluminium alloys in the high-pressure die-casting (HPDC) process, comparing the 1160 mm and 1200 mm positions. Using a comprehensive methodology that combines CT, tensile tests, and SEM, the research demonstrates that the 1160 mm position improves mechanical properties and reduces porosity, with a larger gap at the near-end of the casting, where the yield limit and elongation of the casting increased by 13% and 25% at 1160 mm compared to 1200 mm, respectively. This result shows that appropriate adjustment of the high-speed ram transition position can effectively optimise the organisational structure of thin-walled castings, and then improve their mechanical properties.

## 1. Introduction

The application of the high-pressure die-casting (HPDC) process has made it possible to cast alloy parts with high dimensional accuracy, high productivity, and the ability to produce thin-walled castings with complex shapes [1]. Then, both the high-velocity of the metal (resulting in its turbulence) and the difficulty for air to escape from the mould cavity contribute to the porosity of the castings produced by the HPDC process [2,3,4]. The cause of porosity is mainly due to air entrapment in the molten metal, and the adjustment of process parameters is the main way to control the defects [5]. It has been indicated that changes in pouring temperature, mould temperature, injection rate, and boosting pressure affect the microstructure and, hence, the mechanical properties of casting alloys [6,7,8].

In high pressure die casting (HPDC), the speed at which the ejection punch pushes the molten metal to move is called the ejection velocity. According to the filling state of the molten metal in the mould, the die-casting process is divided into the following three stages: firstly, in the low-speed injection stage, the punch moves slowly, seals the inlet, steadily pushes the molten metal up, and gradually discharges the air inside of the injection chamber; next is the high-speed injection stage, wherein the punch moves at a high-speed, and rapidly fills up the entire cavity of the pouring system with the molten metal in order to break through the resistance of the inner gate and ensure the effective filling speed; lastly, in the boosting stage, the punch’s movement is gradually decelerated to the point of stopping in order to keep the mould pressure inside of the mould. The combination of the stages of this process ensures that the molten metal effectively fills the mould cavity in a short period of time, resulting in high-quality casting. In this case, the position in the press chamber, where the switch from the low-speed to the high-speed plunger stage takes place, is called the high-speed switching starting point [9]. Die-casting materials are generally Al-Si alloys, and Si improves fluidity [10].

Choosing the right process parameters will reduce casting defects. Szalva et al. studied the effect of absolute cavity air pressure on porosity and found that lower cavity air pressure helps to reduce porosity [11]. Dong et al. investigated the effect of super vacuum-assisted HPDC on the repeatability of tensile properties of die-casting alloys, and found that super vacuum-assisted HPDC can improve the tensile strength and plasticity of die-casting alloys [12]. In order to optimise the die-casting process and die-casting process parameters, Ji et al. studied the effects of different die temperatures on the die-casting organisation and mechanical properties of ADC12 aluminium alloy, and found that the optimum value is when the die temperature is 150 °C, and the distribution of dendrites in the hardened surface layer is smaller [13]. Dargusch et al. studied the effect of pressure on the solidification stage and found that porosity decreases with increasing pressure [14]. Niu et al. studied the effect of process parameters on the castability, defects formation, and on the mechanical properties of aluminium alloys in extra-large thin-wall castings, and proposed that the criteria for casting quality should take into account both the geometrical integrity of the casting and the homogeneity of the mechanical properties [15]. Choi et al. investigated the influence of high-pressure die-casting process parameters on high-pressure die-casting performance and mechanical properties by varying the process parameter of different high speeds, and found that the casting performance was optimal at 4 m/s [16].

As J. Lo´pez et al. put it, there exists a critical speed of the plunger, above which, or below which, a very complex liquid flow phenomenon usually occurs in the cavity of the metal–liquid pair [9]. Jiao et al. investigated the microstructural framework of the solidification process and the formation mechanism of the two types of eutectic bands under different die-casting processes, and found that the reduction of the rapid injection velocity accelerated the formation of the central ESC dendritic network [17]. Liu et al. explored the effect of die-casting speed on castings, and found that the size and number of external solidification crystals (ESCs) and pores decreased significantly with the increase in the slow injection speed. At the same time, the distribution of ESCs in the casting samples became more dispersed [18]. The complex flow phenomena can trap air in the liquid metal and eventually lead to a large number of defects in the casting [19]. High-speed ram transition positions can, likewise, lead to complex fluid flow phenomena that can have an impact on porosity and performance, and should be given due attention.

In summary, there are many factors that affect the performance of the casting, vacuum, speed, and so on. This paper explores the effect of high-speed ram transition position on casting performance, provides practical guidance for industrial optimisation, and opens up new perspectives for improving the quality of aluminium castings.

**Figure 1:**
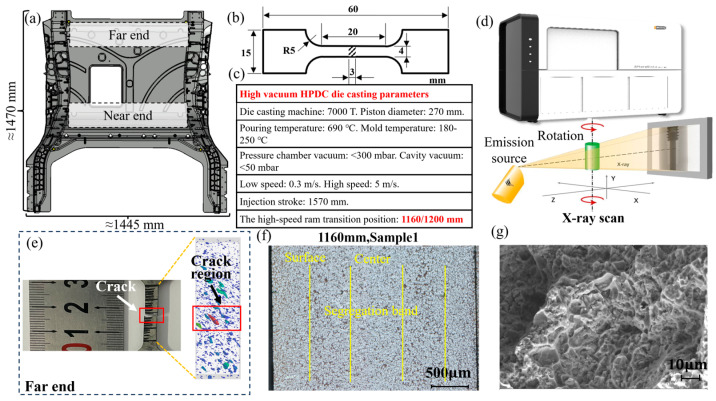
The configuration of (**a**) HPDC casting, (**b**) a testing sample, and (**c**) process parameters. (**d**) X-ray CT scanning equipment and principle. (**e**) X-ray CT scan, (**f**) OM, and (**g**) SEM results.

Therefore, this paper combines X-ray tomography (tomography), tensile tests, metallographic tests, and scanning electron microscopy (SEM) to investigate the effect of the high-speed ram transition position—a process parameter (1160 mm and 1120 mm)—on the mechanical properties and organisational structure of thin-walled castings. Figure 1 depicts the problem-solving methodology, tests, and results.

## 2. Material and Methods

### 2.1. High Vacuum Die-Casting

**Figure 2:**
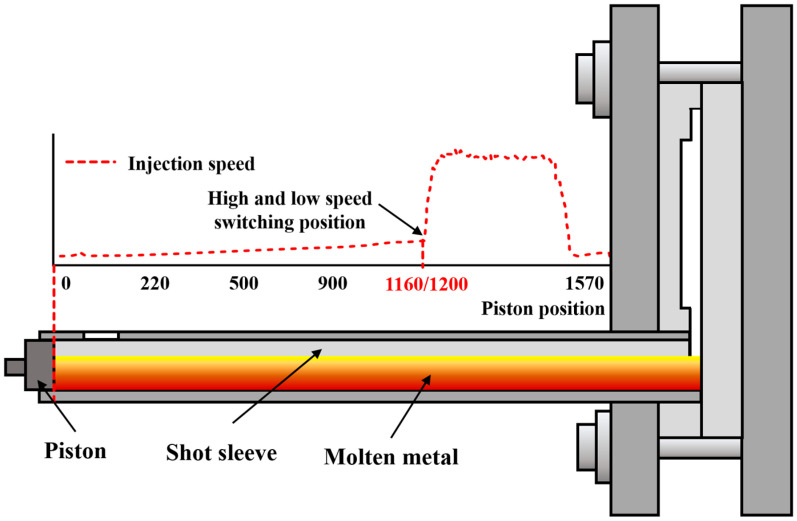
Schematic diagram of high- and low-speed switching points.

A YIZUMI LEAP 7000T horizontal cold chamber die-casting machine (Guangdong Yizumi Precision Machinery Co., Ltd., Foshan, China) was used to produce automotive rear floor parts with overall dimensions of 1445 × 1470 × 590 mm and a piston diameter of 270 mm. The low-speed is 1.24 m/s, the high-speed is 5 m/s, and the speed of the gate is 45.8 m/s. The pouring temperature is kept at 690 °C and the mould temperature is above 200 °C. Aluminium alloys are widely used in casting [20]. The casting alloy material is JDA1b alloy developed by Shanghai Jiao Tong University, a non-heat-treatable die-cast aluminium alloy that combines high strength with excellent ductility [21,22]. The vacuum of the press injection chamber is <300 mbar and the vacuum of the cavity is <50 mbar. The total stroke length of the press chamber was 1570 mm. Therefore, two experiments (at 1160 mm and 1200 mm strokes) were conducted to determine the effect of different high- and low-velocity switching points on the porosity and properties of the castings. Figure 2 shows a schematic diagram of the high- and low-speed velocity switching positions. Subsequently, the walls of the near-end and far-end were cut, respectively, and three samples were cut in each group; the sample sizes are shown in Figure 1b. The samples were labelled as Sample 1 (near gate, 1160 mm), Sample 2 (far gate, 1160 mm), Sample 3 (near gate, 1200 mm), and Sample 4 (far gate, 1200 mm), respectively. The chemical composition of the alloys was determined using an inductively coupled plasma optical emission spectrometer (ICP-OES, PerkinElmer Optima 8300, PerkinElmer, Waltham, MA, USA), and the results are shown in Table 1.

### 2.2. X-Ray CT Scan

In order to quantitatively examine the porosity of the scalar region, see [23]. Three-dimensional X-ray computed tomography (X-ray CT) was performed on a XploreVista 2000 4D (Machine Manufacturer is Microcant Technology (Suzhou) Co., Ltd, Suzhou, China). The tube voltage was 170 kV, the cone beam current was 145 μA, and the resolution was 10 μm. The type and size of the holes were detected according to ASTM E505 [24], ASTM E2422 [25], and T/CASE 301-2023 [26].

### 2.3. Tensile Test

The tensile tests were carried out on an MTS C45.305EEEY electromechanical universal testing machine at a speed of 1 mm/min (MTS Systems Corporation, Eden Prairie, MN, USA). A HAYTHAM SV 1200-32 non-contact signal testing and analysing system (Vitrek Corporation, Lockport, IL, USA) was used for force-displacement measurements. Three sets of samples and tensile tests were carried out at the same position on the castings, at a 1160 mm and 1200 mm starting point at high-speed, to obtain the mechanical property data.

### 2.4. Microstructure Characterization

The specimens were analysed for microstructure and fracture morphology. A Zeiss Imager.M2m microscope (Shanghai, China) was used to observe the microstructure. A Gemini SEM 500 scanning electron microscope (Zeiss, Shanghai, China) was used to observe the fracture morphology of the alloy.

## 3. Results and Discussion

### 3.1. Pore Results

In this study, a detailed internal structural analysis of rear floor specimens of die-cast aluminium alloys was carried out using high-resolution 3D X-ray tomography (Micro-CT). By scanning the proximal and distal specimens at 1160 mm (Sample 1, near-end; Sample 2, far-end) and 1200 mm (Sample 3, near-end and Sample 4, far-end) pressure chamber under high-speed starting conditions, this study successfully identified and classified the three main types of pores in the specimens, which are as follows: air holes, air-shrinkage holes, and shrinkage holes.

**Figure 3:**
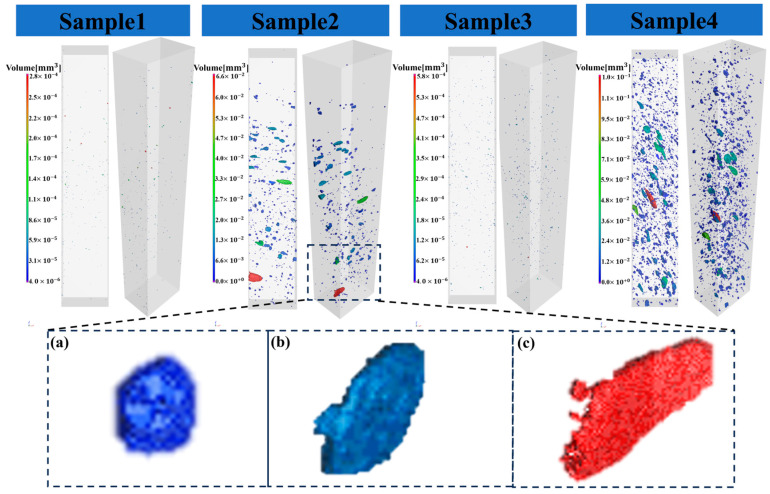
Sample 1, Sample 2, Sample 3, and Sample 4 CT scan results. (**a**) Air holes, (**b**) shrinkage holes, (**c**) air-shrinkage holes.

The formation of air holes is due to gas entrapment during the mould-filling process, as shown in Figure 3a. These pores are formed during the early stages of solidification of the casting, and they maintain a small, relatively rounded morphology, and have a high internal pressure, comparable to the initial pressure. The gas holes are characterised by their small size and are formed during the early stages of solidification of the casting.

Shrinkage holes, on the other hand, are formed during the solidification of the casting due to the inability of the pressure chamber’s pressure to be effectively transferred to the inside of the cavity during the solidification of the inner gate, as shown in Figure 3b. Shrinkage defects will be formed if there are residuals in the hot joints of the casting. The formation of shrinkage holes is related to the pressure drop in the paste zone caused by solidification shrinkage, hydrogen precipitation over percolation, and the nucleation and enlargement of the holes. Shrinkage holes usually form at high solid phase volume fraction stages, and grow only between dendrites, resulting in extremely complex shapes. Gas shrinkage holes, on the other hand, are the result of the interaction between gas holes and shrinkage holes, and their formation is caused by the combination of gas involvement and solidification processes, as shown in Figure 3c. Gas shrinkage holes are often characterised by long tails or other strange shapes. As the solid phase fraction increases, the pressure from the pressure chamber pipe to the mould cavity decreases, and the early holes are compressed by the regular pressure, resulting in a decrease in their internal pressure. The shape of the air-shrinkage holes is more complex than that of the air holes, with a lower degree of roundness.

By comparing the proximal and distal ends of the castings under the same high-speed starting point, it is found that there is a significant difference in the defects contained in the two, with the distal end containing more defects. In addition, there is also a significant difference between the proximal and distal ends of castings with different high-speed starting points, as shown in Figure 3. The CT scan results show that there are fewer porous defects in the proximal and distal ends of castings with a high-speed starting point of 1160 mm, compared to castings with a high-speed starting point of 1200 mm. These findings are important for understanding the effect of different process parameters on the internal quality of the castings.

**Figure 4:**
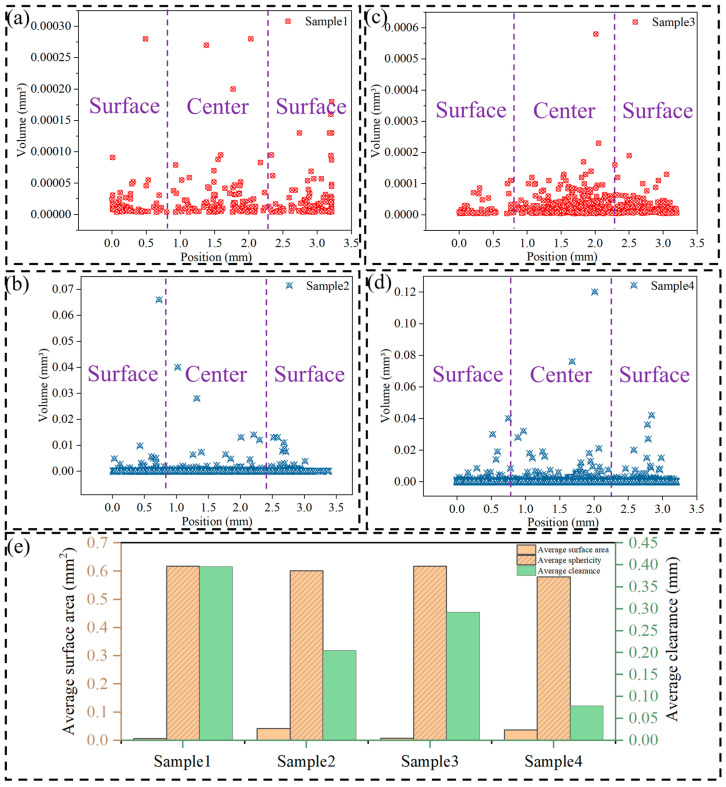
(**a**) Sample 1 pore size distribution in the thickness direction of the sample. (**b**) Sample 2 pore size distribution in the thickness direction of the sample. (**c**) Sample 3 pore size distribution in the thickness direction of the sample. (**d**) Sample 4 pore size distribution in the thickness direction of the sample. (**e**) Statistical analyses of pore information.

Further quantitative analysis of the CT results revealed the microscopic characteristics of the holes. Statistical analyses of the pore volume in the thickness direction, as shown in Figure 4a–d, reveal that the pore volume is more uniformly distributed over the thickness in both the proximal and distal ends in the 1160 mm case, as compared to the 1200 mm case with a high-speed starting point. In tensile testing, a concentrated distribution of pores results in a specimen that is more prone to fracture. As shown in Figure 4e, in the proximal specimen with a high-speed starting point of 1160 mm, the number of holes is low, with an average diameter of 0.048 mm, an average sphericity of 0.616, and an average surface area of 0.005 mm^2^, while in the distal specimen, the number of holes is high, with an average diameter of 0.108 mm, an average sphericity of 0.601, and an average surface area of 0.041 mm^2^. For samples with a high-speed starting point of 1200 mm, the proximal specimen had an average diameter of 0.052 mm, an average sphericity of 0.617, and an average surface area of 0.006 mm^2^; the distal specimen had an average diameter of 0.105 mm, an average sphericity of 0.579, and an average surface area of 0.036 mm^2^.

Therefore, this study suggests that, in the actual production process, the overall performance of the castings can be improved by appropriately adjusting the high-speed starting point of the high-speed ram transition position within the press chamber to reduce the casting porosity, as well as the distribution of porosity in the specimen. A high-speed ram transition position lead to different casting filling processes, which, in turn, affect the quality of the castings. These findings provide a scientific basis for the optimisation of die-casting process parameters.

### 3.2. Mechanical Property Analysis

**Figure 5:**
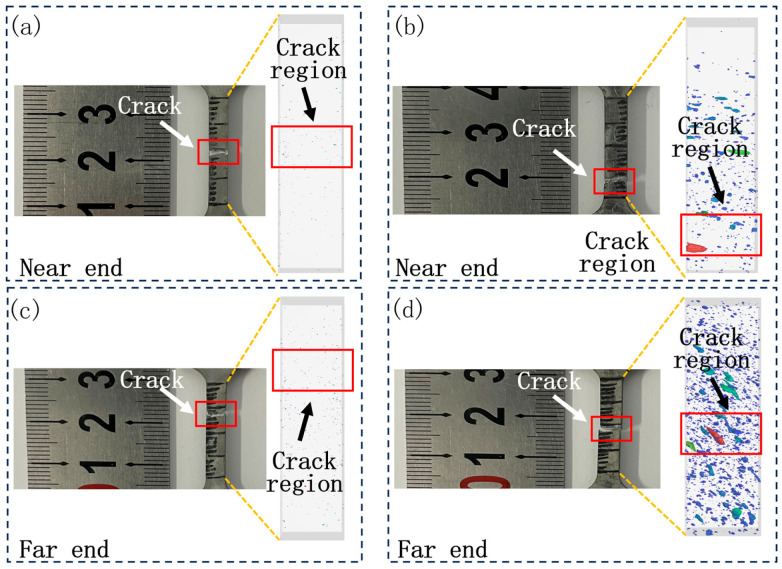
Tensile fracture locations and X-ray CT results: (**a**) Sample 1; (**b**) Sample 2; (**c**) Sample 3; (**d**) Sample 4.

Figure 5 shows the tensile fracture locations and X-ray CT results for several typical specimens. It is easy to find that the area where the holes are concentrated highly coincides with the location of the specimen fracture. Therefore, it can be concluded that the holes are the key factor in initiating the fracture. When there are defects in a part, the load bearing area of the part decreases [5,19]. In the presence of external loads, a multiaxial stress state develops at the hole and induces a strain concentration in its vicinity [20]. This indicates that large-sized holes tend to induce fracture failure in castings, leading to degradation of mechanical properties.

**Figure 6:**
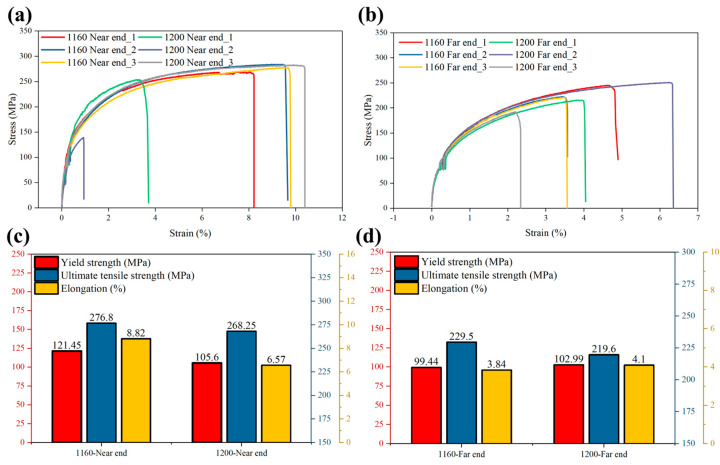
Distribution of mechanical properties at near-end and far-end: (**a**) stress–strain data at near-end; (**b**) stress–strain data at far-end; (**c**) mechanical property statistics at near-end; (**d**) mechanical property statistics at far-end.

The trend of tensile properties with a casting position and different high-speed starting point positions is shown in Figure 6. Among them, there is a sample failure in the proximal end of the casting with a high-speed starting point of 1160 mm. Therefore, the average value of two samples was chosen for all subsequent mechanical property statistics.

As shown in Figure 6c, comparing Sample 1 with Sample 2 in the proximal region of the casting, it is found that Sample 1 exhibits higher mechanical properties. The yield strength was raised from 105.6 MPa to 121.45 MPa, an increase in 13%; the ultimate tensile strength was also raised from 268.25 MPa to 276.8 MPa, an increase in 3%; and the elongation was significantly raised from 6.57% to 8.82%, an increase in 25%. This corresponds to the more uniform pore distribution of Sample 1 than Sample 3 in Figure 4. These data indicate that Sample 1 is better than Sample 2 in all mechanical properties, and the numerical results are consistent with those of Zhang et al., who investigated the tensile test properties of die-cast aluminium alloys [22].

As shown in Figure 6d, in the distal region of the casting, Sample 2 exhibits a slight decrease in yield strength and elongation, from 102.99 MPa to 99.44 Mpa, a decrease in 3%, and a decrease in elongation from 4.1% to 3.84%, a decrease in 6%, as compared to Sample 4. However, the ultimate tensile strength increased from 219.6 Mpa to 229.5 Mpa, an increase in 3%. This change in properties may be attributed to the presence of pores and holes in the specimens that caused stress concentration in the region surrounding them when they were subjected to multiaxial stresses, thus affecting the overall mechanical properties of the material.

### 3.3. Microstructure Analysis

**Figure 7:**
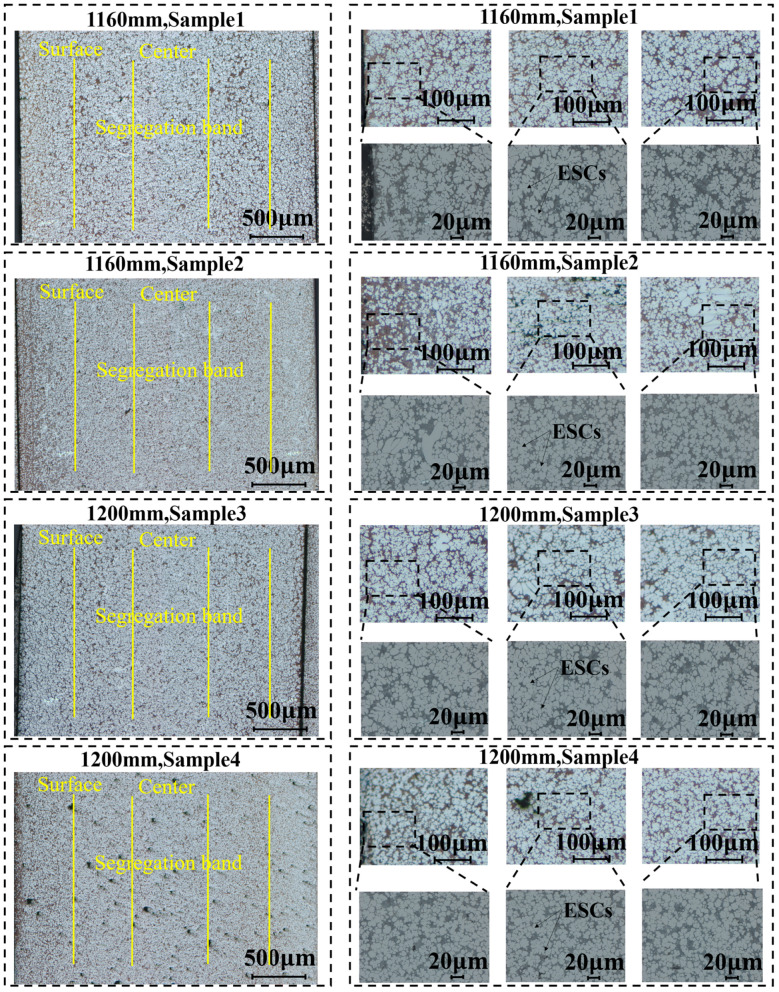
As-cast microstructure of proximal and distal ends at 1160 mm and 1200 mm states.

The microstructures of the castings at the proximal and distal ends of the rear floor at high-speed starting points of 1160 mm and 1200 mm in the press chamber are shown in Figure 7, with significant inhomogeneities in the thickness direction. Firstly, deep segregation bands or defect bands are observed in the range of about 500 to 800 μm from the surface of the samples, and high eutectic Si content is found in the segregation bands. Numerous studies have been carried out to find segregation bands or defect bands in many castings and alloys [8,27,28,29], in which holes or even tears are usually observed, but the defect bands are not obvious. Metallographs show that the die-casting tissue is mainly composed of α-Al matrix (bright white), as well as eutectic tissue (dark grey, consisting of eutectic Si particles with eutectic aluminium) [30]. The α-Al matrix consists of coarse dendritic ESCs (externally solidified crystal) and fine spherical grains (fine cell). Outside of the deviation zone, i.e., within ~200 μm from the surface of the sample, it is called the skin/surface layer, which is mainly characterised by the uniform diffuse distribution of fine α-Al grains and eutectic Si in the surface region, with low ESC content. The part within the deviatoric zone (the central region) has significantly more ESC, which is consistent with the findings of previous studies [31,32,33]. In addition, a higher amount of defect content can be observed within the central region. During the die-casting process, the liquid metal is squeezed by the piston and rapidly enters the cavity, which usually takes 1 to 3 s. During this period, a part of the liquid metal takes the lead in nucleation and growth, and eventually become coarse dendritic ESC; the dendrite length can be up to ~ 100 μm. For the other liquid metals, the higher degree of subcooling causes the rate of nucleation to increase, which results in a large number of nucleation points. Due to the strong shear effect during solidification, uniformly fine-equiaxed grains with a diameter of ~10 μm are formed.

### 3.4. Fracture Surfaces

During high-pressure die-casting of aluminium alloys, the high-speed filling of cavities with metal liquid may cause gas entrapment and produce pore defects, and these pore defects reduce the mechanical properties of castings. SEM analysis of fracture morphology and EDS analysis of local pore locations in the fracture were carried out.

**Figure 8:**
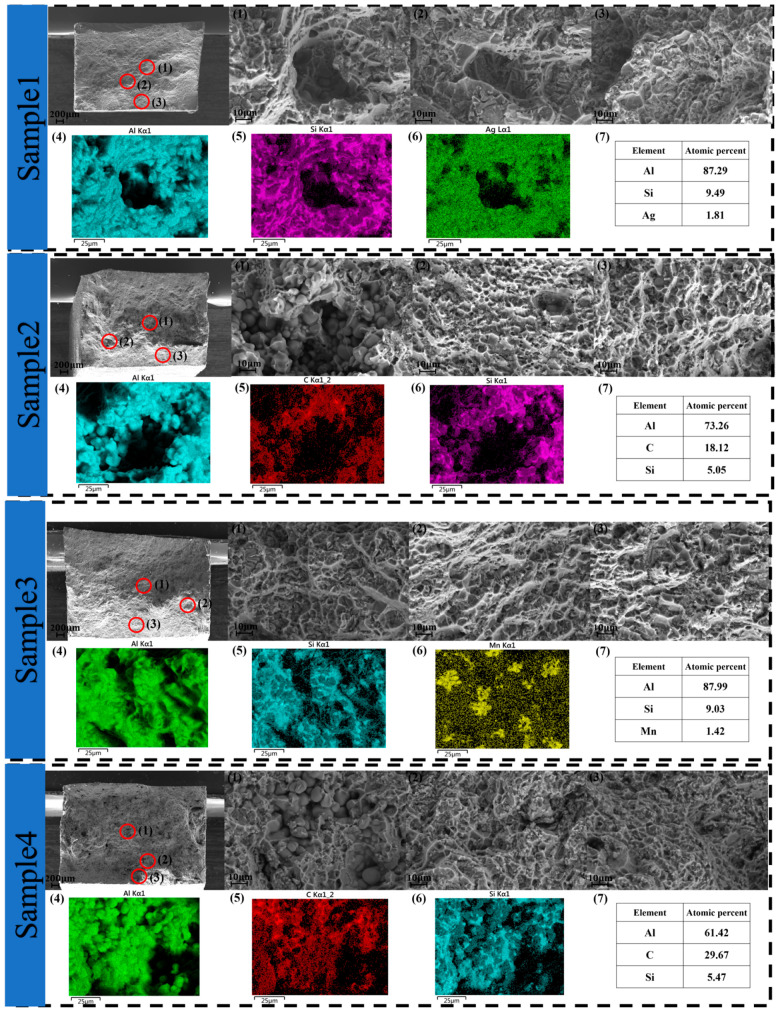
Proximal and distal fracture morphology at 1160 mm and 1200 mm: (1), (2), and (3) are localised morphology of the respective fracture ports; (4), (5), and (6) are the EDS images of the respective fracture (1) position; (7) is the atomic percentage.

In Figure 8 fracture analysis, Sample 2 and Sample 4 have higher porosity and larger pore sizes at the fracture compared to Sample 1 and Sample 3, suggesting that the distal end of the large die-cast aluminium alloy casting will trap more gas. Further comparisons of the proximal fracture morphology at different high- and low-velocity starting points (i.e., Sample 1 vs. Sample 3) demonstrated that the fracture at Sample 3 showed denser and more concentrated tear ridges compared to Sample 3, as well as a significant increase in the number of river-like cleavage surfaces. This phenomenon is consistent with the fracture analysis performed by Wang et al., and is indicative of the occurrence of more brittle fracture characteristics [34]. This phenomenon is consistent with the lower-strength characteristics of the specimen, with a high-speed ram transition position of 1200 mm. When comparing the distal fracture morphology at the transition location of the high-speed ram (i.e., Sample 2 vs. Sample 4), it was found that both exhibited more shear tear ridges and brittle cleavage surfaces. Furthermore, the fracture of Sample 4 also shows more exposed carbon particles. This phenomenon is consistent with that encountered by Yang et al. when analysing the ports [35]. Usually, the carbon particles in the specimen have a good bond with the surrounding matrix; however, under the action of external forces, these carbon particles are prone to becoming a weak region of stress concentration, which, in turn, triggers the emergence and expansion of cracks. Crack formation is mainly caused by the sprouting and expansion of a large number of micropores under tensile stress, which ultimately leads to the fracture of the material. Therefore, the degree of exposure of carbon particles and their bonding condition with the matrix may be important factors influencing the fracture behaviour.

In the EDS image analysis, the distribution of Al and Si atoms is approximately the same, which is due to the fact that Si forms a eutectic organization with the Al matrix [11,12]. The large amount of silicon content produces a eutectic organization that provides a lower melting point and lower shrinkage. Silicon is likely to cause river-like cleavage surfaces on the fracture, which can easily lead to a failure fracture of the material. We can notice the presence of Ag in the proximal Sample 1, as shown in Figure 8 Sample 1 (6), where Ag atoms are evenly distributed in the fracture. Ag was also detected in the fracture table analysis of die-cast aluminium alloy by Yang et al. [36]. This is due to the very fast cooling rates that usually accompany high-pressure die-casting processes. During this rapid cooling process, the rate of diffusion of elements in the alloy is inhibited, which means that less soluble elements in the alloy (e.g., Ag) may not precipitate out in time to form large particles of precipitates. Instead, it remains in a more homogeneous solid solution, and eventually Ag is solidly dissolved in the aluminium alloy matrix in a more uniformly distributed form. Mn is enriched in localised areas in Sample 3. This is due to the fact that the solidification characteristics of Mn are different from those of Al, which may be formed by the phenomenon of segregation. At the distal end, the three most abundant atoms appear to be Al, Si, and C. The distribution of element C corresponds not only to more carbon particles in the fracture analyses of the Sample 2 and Sample 4 fractures, but also to worse mechanical properties of distal Sample 2 and Sample 4 in the mechanical property tests. This is due to the presence of gas entrapment or inclusions in the vicinity of the pores, which prevented the magnesium from being adequately distributed in the vicinity of the pores.

It is unreasonable that the high-speed ram transition position can lead to higher porosity, which, in turn, becomes the main reason for the degradation of mechanical properties of aluminium alloy die-castings. Therefore, optimising the high-speed ram transition position is a key measure to improve the internal defects of HPDC aluminium alloy and enhance its mechanical properties.

## 4. Conclusions

In this paper, high vacuum die-casting experiments were conducted by setting different high-speed ram transition positions. Combined with X-ray CT, tensile test, and microscopic characterization methods, the porosity, mechanical properties, and microstructure of large and complex thin-walled one-piece die-cast aluminium alloy parts are analysed. The following conclusions can be drawn from the study:

(1) Statistical analysis of the CT results shows that, in the thickness direction, the specimen with a high-speed slide transition position of 1160 mm has a more uniform pore distribution compared to the specimen of 1200 mm.
(2) The results of the tensile test show that, compared to the specimen with a 1200 mm transition position of the high-speed slider, the yield limit of the proximal mechanical properties of the 1160 specimen is increased from 105.6 MPa to 121.45 MPa, which is an increase in 13%, and the elongation is increased from 6.57% to 8.82%, which is an increase in 25%.
(3) Analysis of the fracture morphology of the die-cast aluminium alloy showed that the 1200 mm specimen exhibited denser tear ridges and more river-like cleavage surfaces in the proximal fracture compared to the specimen with a high-speed slide transition position of 1160 mm, presenting more brittle fracture characteristics, which is consistent with the lower-strength properties at this condition.
(4) EDS image analysis reveals the presence of fine torn grains and a large number of silicon particles in the Al matrix lead to the formation of a eutectic organization that may form river-like cleavage surfaces on the fracture, leading to a failure fracture of the material. Excessive carbon or carbide precipitation leads to grain boundary embrittlement and accelerated crack extension, thus increasing the risk of fracture.

## Figures and Tables

**Table 1:** Composition of parts (wt.%).

Al	Si	Mn	Mg	Ti	Fe	Other
Bal.	6.83	0.56	0.27	0.12	0.097	<0.1

## Data Availability

The raw data supporting the conclusions of this article will be made available by the authors on request due to [Confidentiality of enterprise projects].
